# What is the performance in public hospitals? A longitudinal analysis of performance plans through topic modeling

**DOI:** 10.1186/s12913-021-06332-4

**Published:** 2021-04-09

**Authors:** Guido Noto, Andrea Carlo Lo Verso, Gustavo Barresi

**Affiliations:** 1 Department of Economics, University of Messina, Messina, Italy; 2 Department of Management, University of Bologna, Bologna, Italy

**Keywords:** Performance, Public management, Hospital, Healthcare, Topic modeling

## Abstract

**Background:**

Both the concept of performance and the role of hospitals in health systems evolved significantly in the last decades. Today, the performance in health could be defined as the ability to create ‘population value,’ and the hospitals’ role is to support this aim by providing acute care and by integrating and coordinating their activity with other settings of care. This research aims to assess how and with what degree the management of public hospitals have embraced in practice the updated concept of performance and their new role.

**Result:**

The paper analyses 181 performance plans of 48 Italian autonomous public hospitals over a nine-year period through the topic modeling algorithm called Latent Dirichlet Allocation (LDA). This is a method that allows for analysing large textual corpora that generates a representation of the latent topics discussed therein. The concept of performance in public hospitals was framed into 15 topics resulting from the analysis of the hospitals’ performance plans. The prevalence of each topic was analysed through the period considered so as to understand the evolution of performance-related practices over the last decade.

**Conclusion:**

In recent years, the concept of performance in hospitals evolved toward the adoption of an outcome-based and population-based perspective. Additional effort should be devoted toward improved collaboration and integration of care with other settings.

**Supplementary Information:**

The online version contains supplementary material available at 10.1186/s12913-021-06332-4.

## Introduction

The concept of performance was extensively introduced in Public Administration with the adoption of New Public Management (NPM) reforms [1]. Originally, according to the NPM paradigm, the performance was mainly intended as the capability of public organizations to pursue efficiency and increase productivity in the delivery of public services [1–3]. In the last decades, however, the concept of performance has significantly evolved through the emerging wave of the so-called post-NPM initiatives [2]. In particular, public management scholars today agree that performance should not be focussed exclusively on the input and the output produced by single public organizations – i.e. the result of an effort toward greater efficiency and productivity – but should be mainly conceived as the broader outcome achieved in partnership with a wide set of stakeholders for the benefit of the wider community [4, 5].

The healthcare sector has not escaped the above-described trend [6–10]. Particularly, due to changes in the epidemiological context that characterize modern societies, public hospitals have been called, in recent years, to increasingly focus on outcomes at the population level and to integrate their actions with the other providers and stakeholders of the healthcare sector [11].

Both the renewed concept of performance and the new role of hospitals in health systems are quite consolidated in literature, however, it is difficult to assess how and with what degree the management of public organizations have embraced them in practice. Thus, the research questions this contribution aims to answer are: how is performance defined, conceived, and interpreted in public hospitals practice? To what extent public hospitals embraced and are embracing the theoretical paradigms emerging in the literature with regards to performance?

In order to investigate the renewed concept of performance and its adoption trends, the paper analyses the performance plans of a wide number of public healthcare organizations in Italy through topic modeling. These management reports are produced annually and are publicly disclosed. The aim of the management reports is to define the performance targets to be achieved by the organization and its employees. More precisely, the dataset comprises 181 reports published by 48 autonomous public hospitals between 2011 and 2019, counting 9162 pages overall. Topic modeling allows for analysing this large textual corpus through an algorithmic procedure that generates a representation of the latent topics discussed therein [12]. The advantage of this technique is that it joins the human ability to interpret the semantic content of topics with precise quantification of how much any given topic is prevalent within each unit of analysis (e.g., a document, a paragraph, a page).

Italy represents an interesting case to study since it has introduced the first NPM reforms in the early 1990s [7, 13, 14]. In particular, the Italian National Health System (INHS) is a regionally based universal coverage system which adopts the Beveridge financing model [15]. Since the early 1990s, under the NPM wave, legislative reforms have gradually transferred political, administrative, fiscal, and financial responsibilities from the national government to regional authorities [15]. Each regional authority receives funds from the central government and defines its own health plans and strategies. Regional authorities provide health services through: i) Local Health Authorities (LHAs), i.e. geographically based organisations financed by capitation, which deliver public health, community health services, and primary care directly as well as secondary and specialist care through directly managed facilities, or by purchasing services from public hospital institutions or private accredited providers; ii) autonomous public hospitals focused on acute care and financed by service tariffs; and iii) private providers (accredited with the INHS) financed by service tariffs. This research focuses on autonomous public hospitals.

As in other countries, NPM reforms were gradually substituted by post-NPM initiatives. The last reform that could be labelled as an NPM initiative was introduced in 2009 (see. Law 150/2009, also known as “Legge Brunetta”) and was mainly oriented at introducing the practice of performance evaluation at both individual and organizational level [16] through the adoption of performance plans that public organizations are required to prepare, update, and publish every year. The decision to focus on public hospitals is due to the fact that in 2015 a ministerial decree (DM 70/2015, also known as “Decreto Balduzzi”) was published in order to guide public hospitals in changing their role and organizational structure toward better integration with the regional health system in which they operate. This could be labelled as a post-NPM initiative that also accounts for the evolved role of public hospitals.

The article is structured as follows. The next section reports a review of the literature on the evolving role of hospitals with a focus on performance-related aspects. The third section presents the methodology adopted and how this has been applied to the research. The section that follows reports the evidence emerging from the analysis. Last, discussions and practice implications are presented.

## Theoretical background

The literature on performance in health has been mainly tackled through the lenses of performance management and measurement [10]. The seminal paper dealing with performance measurement in health has been written by Donabedian [17]. This has been extensively re-called even by most recent literature [18–21]. In this contribution, the author focuses on three levels of analysis, namely structure, process, and outcome. The latter considers the most frequently used indicators of quality of medical care (e.g. 30-day mortality indicators, re-admissions, etc.) and concerns the impact that health care has on the health status of patients. The process domain, instead, aims at measuring whether the output of care processes is achieved. Such processes can be considered as intermediate results to be addressed in order to achieve end-results. Lastly, the structure domain concerns the adequacy of resources (financial, human, and equipment) needed to foster processes and, therefore, outcomes.

In the healthcare sector, performance measurement has been largely focussed on the hospital setting since, on average, it accounts for between 22 and 42% of the total national healthcare funds [22], and it focuses on services and activities that are easily measured if compared to other settings such as prevention or primary care. In every health system, hospitals are in charge of the care and management of acute episodes. Since the implementation of NPM reforms, public hospitals have been provided with performance management tools aimed at fostering their processes in terms of productivity and efficiency in delivering acute care and ambulatory services [6, 23, 24]. In this historical phase, hospital performance was conceived and measured as the ability to produce as many treatments as possible with the available resources and to increase financial margin [25, 26].

This way to conceive performance may have provoked unintended consequences [27–30] – e.g., delivering non-appropriate but profitable services – mainly related to the strengthening of a “silo” logic [31] according to which public hospitals are conceived as autonomous organizations in competition with other health organizations operating in the same national health system [25].

To overcome this issue, in the first decade of the XXI century, the concept of healthcare performance has been re-defined as the ability to create value [32–34]. Porter [32] identifies value as the relationship between the actual patient health outcomes and the cost of treatments. Thus, according to this definition, hospitals create value when they treat acute episodes of patients achieving good outcomes and containing costs when compared to alternative solutions.

In recent years, healthcare organizations have been exposed to an increased complexity deriving from changes in epidemiological conditions – such as population ageing– which are determining new care needs mainly related to the increasing emergence of chronic diseases. Due to their mission, hospitals cannot fully address chronic patients’ needs which are continuous and do not decrease over time. In fact, in these cases, health systems goals are not exclusively related to the delivery of good services or treatments, but to maintain the best possible quality of life for patients and to provide continuous care. Dealing with these emerging needs requires collaboration between different professionals operating in different organizations and care settings [26, 35]. Due to that, public hospitals all over the world have been called to integrate and coordinate their activity with the other organizations and providers of the health systems in which they operate [11, 36, 37].

The reference theoretical paradigm supporting this change is the ‘population value’ one [38, 39]. According to this paradigm, value does not exclusively correspond to the achievement of better patient outcomes with the available resources but is the ability of the health system to provide care to the people that could benefit most from it [38–40]. A health system creating value is thus a system in which the people receiving care are those who could benefit the most from it [26, 39]. As a consequence, new strategies and actions have been designed and implemented to guide the evolution of hospitals’ role toward improved coordination, integration, and continuity of care [37, 41].

## Method

The research design consists in the application of text-analysis to a dataset comprising all of the available performance plans of Italian autonomous public hospitals from 2011 to 2019 (see table in the Additional file 1). Performance plans are administrative documents prepared by the top management of each Italian public organization. These identify the organizational objectives and define the indicators to be used to measure, monitor, and evaluate organizational performance. These indicators are also adapted to the organizational structure and thus attributed to each organizational unit and individual. In particular, the national guidelines to prepare performance plans identify four building blocks to be developed: i) the mission, organization, and personnel of the administration; ii) the three-year objectives, indicators, and targets; iii) the annual objectives, indicators, and targets; iv) the translation of the organizational objectives into individual ones.

A total of 181 performance plans, amounting to 9162 pages and 2.8 million words, have been collected from the institutional websites of 48 autonomous Italian public hospitals over the period 2011–2019.

A pre-processing step is always needed in text-analysis to clean up the corpus. In this case, following a consolidated practice, the authors removed Italian stop words (i.e., highly frequent words that convey little meaning such as “the”, “and”), very infrequent words (recurring less than three times), words shorter than three characters, punctuation, and numbers. After this procedure, the text corpus comprises a total of almost 1.5 million analysable words. Given this large amount of textual data, the authors decided to resort to unsupervised text-analytical techniques in order to distil the thematic nuclei addressed therein. In particular, data analysis is performed through topic modeling: an algorithm-based technique originally developed in the field of computer science and natural language processing and increasingly adopted in management studies. Differently from other automated content analysis procedures, this method generates a list of topics recurring in large textual corpora without the aid of predefined, explicit dictionaries or interpretive rules [12]. The most widely adopted topic modeling algorithm, Latent Dirichlet Allocation (LDA henceforth), is based on the idea that documents are represented as random mixtures over latent topics, and each topic is characterized by a distribution over words [42]. The output of a topic model generated through LDA thus comprises a set of clusters (topics) of words consistently co-occurring in units of text and the distribution of these topics over the entire textual corpus. This yields a quantitative measure of the extent to which every single word is associated with any given topic, and of the weight (i.e. prevalence) that each topic has in any given unit of text.

Topic modeling is thus a technique that joins a purely inductive, data-driven quantitative text-analysis with an interpretative endeavour of the analysts who attribute meaning to the topics [12, 43]. The only parameter which is a priori imposed on the model is the number of topics. Although some quantitative techniques to evaluate the quality of topic models have been developed in the past years, the selection of the right number of topics in terms of their interpretability is usually based on the analysts’ capability to attribute meaningful labels to each topic and to the desired granularity of the overall model [44].

After having run models with 12 to 25 topics, the authors decided to base the analysis on the 18-topic model. This choice has been reached after a careful inspection of each model aided by the visualization tool LDAvis [45], which displays through multidimensional scaling the proximity among topics in terms of their co-occurrence in documents and, as a proxy, in terms of their semantic similarity. Furthermore, the appropriate interpretation and labelling of each topic’s content have been achieved through multiple stages of comparison of each author’s own understanding of the words composing the topics and by constantly referring to the original texts. A common practice in topic modeling is to focus the analysis onto a certain subset of topics, as some topics’ meaning may result as indeterminate [46]. Since three topics were made of rather uninformative combinations of words, they have been discarded from the following analysis. As a result, the analysis focussed on the remaining 15 topics.

This initial interpretative effort has been performed by following a purely inductive approach aimed at defining the topics with descriptive labels. In a subsequent phase of analysis, some of the emerging meanings attached to each topic have been observed to match with the theoretical paradigms outlined in the previous section such as population value, performance management and collaborative governance. Thus, the inductive topic analysis has been imbued with further meanings by grouping clusters of topics according to theoretical categories derived from the literature.

Finally, in order to appreciate the dynamics of performance plans’ content, the authors grouped the 15 topics also on a temporal basis (i.e., by triennia), gauging how the aggregate prevalence of each topic across documents varied from time period to time period. More precisely, the prevalence metrics (i.e., how much a given topic weights in the composition of a textual unit) has been averaged over the total number of pages of performance plans issued in each triennia. To better explain the sample analysed, a table which reports the pages of the documents per hospital and region was included in the Additional file 1.

## Results

As mentioned in the previous sections, the topic model applied to these documents identified fifteen (15) different topics that characterize their content. These are displayed and labelled in Table 1.

**Table 1:** Topics (firsts 20 words)

Topic 1: Performance cycle	Topic 2: Performance evaluation	Topic 3: Vision & Mission	Topic 4: Network and care pathways	Topic 5: Strategic analysis	Topic 6: Legal framework	Topic 7: Identity	Topic 8: Governance structure	Topic 9: Processes	Topic 10: Eco-Fin resources	Topic 11: Outcomes	Topic 12: Specialized care structure	Topic 13: Corruption risk prevention and transparency	Topic 14: Population and demographics	Topic 15: Human resources
Performance	targets	organization	activities	organization	regional	organization	direction	performance	balance	admissions	surgery	transparency	population	staff
Targets	rating	Research	area	activities	plan	activities	director	DRG	costs	days	medicine	corruption	years	total
Plan	budget	activities	patients	analysis	health-based	university	general	activities	cheap	within	department	prevention	year	role
Strategic	performance	development	management	points	law	regional	management	admissions	goods	year	clinic	plan	province	senior executives
Organization	structure	services	paths	to be	companies	hospital-based	organizational	stay	expense	intervention	illnesses	performance	illnesses	sector
Programming	process	quality	network	structures	region	of hospital	activities	recovery	services	performance	unit	measures	data	leadership
Operating	organizational	through	pathway	context	hospital	university	structures	regime	administrative period	value	therapy	activities	organization	health-based
Process	achievement	resources	plan	internal	organization	Research	department	year	cost	interventions	urgency	careggi	rate	time
Cycle	individual	support	integration	hospital	decree	polyclinic	organization	number	value	surgical	area	three-year	age	technical
Indicators	system	health	particular	system	national	reference	control	day	resources	standard	general	pag	region	year
Rating	year	performance	scope	such	health	region	staff	outpatient	year	volume	pediatric	anticorruption	residents	years
Management	results	training	development	part	system	Hospital	health	average	organization	indicator	center	administration	total	professional
Reference	direction	respect	Hospital	level	resolution	location	departments	total	management	appropriateness	cardiology	execution	inhabitants	employees
Resources	management	integration	project	resources	health	network	resources	ordinary	production	indicators	intensive	integrity	regional	administrative
Measurement	sheet	improvement	interventions	organizational	service	center	administrative	ordinaries	investments	mortality	sod	organizational	equal	employee
Consistency	staff	system	reorganization	always	Article	catania	programming	bed	revenues	set off	transplants	risk	about	doctors
Strategic	control	innovation	utilization	strenght	regional	aou	structure	reduction	health	stay	medical	ref	index	health
Organizational	organization	processes	appropriateness	whose	provisions	functions	administrative	value	health-based	dRG	laboratory	updating	table	leader
Context	organizational	organizational	patient	opportunity	organizational	health-based	functions	outpatients	result	improvement	emergency	lgs	respect	number
Improvement	phase	to guarantee	areas	Hospital	matter	support	organization	weight	plan	mes	vascular	publication	mobility	area

The topics identified have been clustered into 6 macro-categories. The first category is related to the normative and regulatory aspects of the performance assessment and evaluation in Italian public organizations. This includes the topics 1, 2, 6 and 13 labelled “Performance cycle”, “Performance evaluation”, “Legal framework”, “Corruption risk prevention and transparency”.

“Performance cycle” identifies content related to the functioning of the performance cycle assessment according to the Italian Law 150/2009 requirements.

“Performance evaluation” topic is focussed on the criteria established to evaluate individual staff performance, namely how organizational performance is declined at the individual level.

“Legal framework” is a topic characterized by those words representing the legal framework characterizing the performance assessment cycle and the laws and decrees which guide organizational and individual behaviours.

“Corruption risk prevention and transparency” is one of the topics introduced by the Italian Law 150/2009 together with the performance cycle assessment. This focuses on the requirements public organizations in Italy should comply with in order to prevent corruption and to disclose information to the public.

The second category includes strategic elements which characterize public hospitals. This is composed of the topics 3, 5 and 7, i.e. “Vision and mission”, “Strategic analysis” and “Identity”.

“Vision and mission” topic refers to those parts of performance plan documentation in which the vision and the mission of the hospital are defined and made explicit. As previously anticipated, this is one of the content that the law and the related decrees require in the preparation of the performance plan.

“Strategic analysis” groups the part of the documents in which the analysis of the internal and external environment is performed. This is often carried out through the application of the SWOT (Strengths, Weaknesses, Opportunities and Threats) matrix.

“Identity” topic refers to the content in which the organization presents itself to the wide range of stakeholders. In this topic emerges an important role of these hospitals as training and research centres.

A third category considers the topics which refer to the governance and organizational settings. These are “Governance structure” and “Specialized care structure”. “Governance structure” topic is related to the definition and description of the different bodies characterizing the hospital management, e.g. directors, auditors, etc.; while “Specialized care structure” lists the departments or units focusing on specific specialities such as surgery, cardiology, emergency, paediatrics and so on.

These first three categories are the most stable in terms of content, meaning that they refer to parts and sections of performance plans which are often repeated over the years.

The fourth category includes topics 4 and 14 which focus on the integration of hospital activity with the health system in which they operate and the reference population. These are here named “Networks and care pathways” and “Population and demographics”.

“Network and care pathways” is a topic focussed on the organization and management of care pathways for which the hospital plays a key role (chronic diseases, maternal pathway, oncology, etc.). In this sense, it represents the effort of the hospitals toward better integration and collaboration with the other stakeholders operating in the health system.

“Population and demographics” refers to the population health status and characteristics of the territory in which the hospital operates. It represents the willingness of the hospitals to embrace a population perspective when planning their activity.

A fifth category is related to the resources that Donabedian taxonomy would consider as part of the structures. The related topics are 10 and 15, i.e. “Economic and financial resources” and “Human resources”. “Economic and financial resources” groups all the contents referred to the balance sheet such as revenues, expenditures, investments, and other financial measures. “Human resources” topic refers to the typology and numerosity of staff and professionals working in the hospitals, and to recruitment policies.

Two other topics - i.e. 9 and 11 - characterize the performance indicators category. These are “Processes” and “Outcomes”. Both topics are mainly composed of words and expressions coming from the description and formulation of the hospitals’ management’s performance indicators. “Processes” groups those indicators which measure healthcare processes. As such, these indicators are mainly focused on outputs and productivity. “Outcomes” refers to those indicators which are aimed at measuring the outcomes of care. These outcomes include mortality indicators and other outcomes proxies such as caesarean sections, etc.

The identification and classification of the above-described topics characterise the way in which performance is actually conceived by public hospitals in Italy at the managerial level. In particular, the topics classified in the strategy, integration, structure and performance indicators categories are the ones that better describe the concept of performance; while regulation and organizational settings categories are related to the formal and static dimensions of performance.

A longitudinal analysis of the prevalence of the identified and previously described topics allow us to understand how the concept of public hospital performance evolved in Italy in the period considered. In particular, the topics which are most relevant for our analysis are the ones related to integration, structure and performance indicators – i.e. “Network and care pathway”, “Population and demographics”, “Economic and financial resources”, “Human resources”, “Processes” and “Outcomes”.

In order to deal with the different distribution of data available, results have been grouped into three triennia: 2011–2013; 2014–2016; 2017–2019. This choice is related to the need for managing a set of data in which some years were underrepresented.

**Fig. 1:**
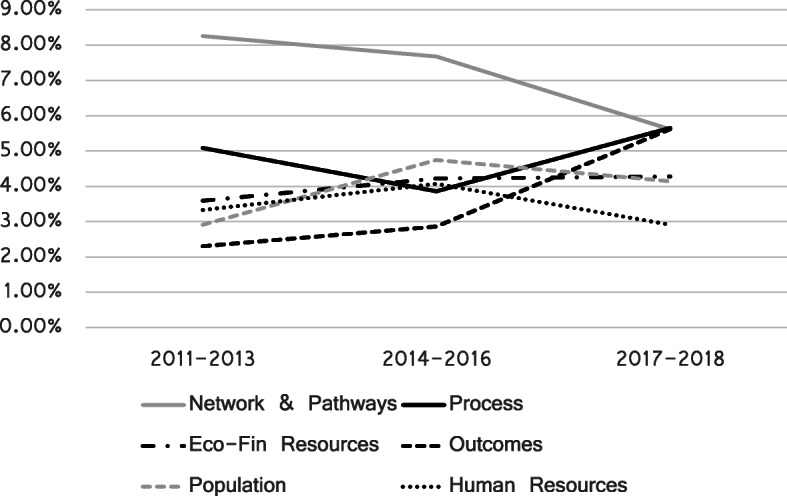
Topics’ prevalence dynamic

Figure 1 displays the prevalence of these topics over time aggregated by triennia.

Starting from the most relevant topic in terms of relative weight, we may notice that “Network and care pathways” topic, although maintaining a relatively high relevance, decreases in prevalence from 8.25% – in the first three-year period – to 5.62% in the last one.

“Process” topic overall increases its prevalence from 5.09% in 2011–2013 to 5.65% in 2017–2019 – despite a slight downturn in the second triennium.

“Economic and financial resources” topic shows a slight improvement from 3.59 to 4.28%, while “Human resources” slightly decreases in prevalence.

“Population and demographics” topic experiences an interesting growth between the first and the second trienniums and remains more or less constant in the third one.

Last, “Outcome” significantly increases its contribution, becoming one of the most significant topics analysed in the last period even though it was one of the less prevalent during the first years considered in the analysis.

In order to provide a comprehensive view of the topics dynamics, the results of all topics are reported in Table 2.

**Table 2:** Topics dynamics

Topic	Category	2011–2013	2014–2016	2017–2019
1: Performance cycle	Regulation	15.01%	13.58%	11.14%
2: Performance evaluation	Regulation	11.96%	10.01%	9.57%
3: Vision & Mission	Strategy	9.19%	10.23%	8.71%
4: Network and care pathways	Integration	8.25%	7.68%	5.62%
5: Strategic analysis	Strategy	8.22%	6.18%	5.88%
6: Legal framework	Regulation	3.22%	4.65%	5.39%
7: Identity	Strategy	6.94%	4.82%	4.06%
8: Governance structure	Organizational settings	5.77%	5.51%	4.09%
9: Processes	Performance indicators	5.09%	3.86%	5.65%
10: Eco-Fin resources	Structure	3.59%	4.22%	4.28%
11: Outcomes	Performance indicators	2.31%	2.86%	5.62%
12: Specialized care structure	Organizational settings	5.20%	4.41%	4.20%
13: Corruption risk prevention and transparency	Regulation	1.00%	4.11%	3.29%
14: Population and demographics	Integration	2.91%	4.75%	4.15%
15: Human resources	Structure	3.33%	4.07%	2.91%

## Discussions

Performance is a dynamic concept that evolves along with individual, organizational, and societal changes. Thus, defining performance makes sense if related to a specific type of unit (e.g., an organization) at a specific time. The approach used here to explore the concept of performance in autonomous public hospitals adopts this perspective. In particular, the empirical analysis concerns a set of organizations that share a mission, role, and institutional environment.

The identification of the topics made here cannot ignore the changes and evolution that occurred in the public sector and the health sector in the last decades. This is the reason why the theoretical background adopted by the authors refers to the key theories and paradigms well established both in public management and healthcare management literature.

First of all, a framework that was key in order to support the authors in the categorization of the topics describing performance in health is the Donabedian one [17]. Through these lenses, it was possible to connote those topics related to structures, processes, and outcomes. In particular, the topics describing structure were two: one focussed on economic and financial resources, which remains more or less stable over time, and one related to human resources, which experienced a decrease in prevalence in the last triennium. This may be related to the Italian legislators’ constraints on human resources recruitment, which reduced the decision powers of regional health systems and consequently of public hospitals [30].

The topic related to process measures of performance always represented a pillar in the assessment of hospital performance due to their measurability in terms of outputs and the importance given to efficiency and productivity inherited by NPM reforms. This is demonstrated by the rising importance of the topic over the period considered.

What is of particular interest is the dynamic characterizing the “outcome” topic. Although poorly represented at the beginning of the period analysed, outcomes become particularly relevant to represent performance in the following years until becoming as much represented as processes. This important result confirms that practice adopted the multiple suggestions deriving from public management literature, which, in the last decades, highlights the importance of measuring outcomes when assessing and managing organizational performance [4, 8, 47]. The increasing relevance of outcome could also be motivated by the introduction in the early 2010s of national, regional, and inter-regional programmes to measure health care performance across the healthcare providers of the Country such as the National Outcome Evaluation Program (Programma Nazionale Esiti – PNE) developed by the Italian National Agency for Health Services (Agenzia per Nazionale per i Servizi Sanitari - Age.Na.S.) and the Inter-Regional Performance Evaluation System developed by the Management and Health Laboratory of Sant’Anna School of Advanced Studies [48].

The other theoretical framework adopted by this research is the population value one [38]. Population value combines the concept of value in health as defined by Porter [32] with a population perspective. A population perspective encompasses whole populations (i.e., defined by geography, insurance coverage, or attribution) and not only those with specific illnesses or needs [10, 49]. In this sense, hospitals are called to consider the impact of their activity on the population they are directly or indirectly serving. In this sense, the growing attention of performance plans on the epidemiological characteristics of the population served by the hospital may demonstrate the progressive adoption of such a population perspective – see “Population and demographics” topic dynamic.

What shows a countertrend with regards to the literature discourse on performance in health is the dynamic characterizing the “Network and care pathways” topic. As one may notice from Table 2, although it remains one of the key topics addressed by hospitals’ performance plans, the relative weight of this topic decreases from 8.25%, in the first triennium, to 5.62% in the last one. This result is surprising because, in the last decade, both public management [50, 51] and healthcare management literature [11, 35, 37, 41] emphasized the importance of the concept of collaborative governance, networks, and integrated care. Moreover, in Italy, the Ministerial decree nr 70 of 2015 aimed at translating some of these concepts into practice and health system regulation. Such a result could be explained in two ways. One explanation could refer to an implementation gap currently existing between what literature suggests and what practitioners do. Although this explanation could be considered valid – acknowledging that the theory-practice gap exists in almost all social sciences and disciplines – it does not stand if we consider that Italian law already aimed at fostering networks and care pathways. Another explanation could be based on the hypothesis that this theoretical paradigm shift has not been supported by performance plans, but other management tools have been employed to address it. Moreover, it is also important to remark that this decreasing trend is expressed in relative terms. A decrease of the same entity was also experienced by other “stable” topics such as “Performance cycle” or “Performance evaluation” which refers to parts of the documents that do not change much over the years because of their compulsoriness (see Table 2). Therefore, the attention given to this topic by performance plan could be considered as stable in absolute terms and such a decrease could be attributed to the fact that performance plans, since their adoption, are broadening their scope and content. In fact, the average number of pages increases from 39 – in the first triennium – to 55 in the last one (see Additional file 1). This demonstrates that performance plans are becoming an effective management tool to support the designing of a performance management system from the definition of strategies to the identification of performance targets and the evaluation of results. In the first years of adoption, this tool, introduced by the Italian law 150/2009, was mainly prepared so as to comply with a legal requirement and, in the first triennium analysed, the most relevant topics in these plans were those related to compulsory contents. Through the years, other topics referred to the management and measurement of performance emerged, such as the “outcome” and the “population and demographics” one.

The results obtained and related to the evolution of the concept and the dimensions used to assess and address performance, confirm a global trend according to which performance is conceived as a multidimensional construct comprehending structure, process, and outcome measures at both the institution and inter-institutional or population level [20, 52–54]; hospitals are thus called to measure and manage performance consistently [24, 55].

The adoption of topic modeling techniques revealed to be an effective solution to understand management decisions and processes when these are reported in documents. In fact, differently from other content-analysis techniques, this methodology does not require the adoption of predefined dictionaries by implementing a purely bottom-up process for uncovering texts’ semantic structure that, being independent of human coders, is not constrained to the analysis of small-scale samples. In this way, the method used here yields an analysis that preserves the overall semantic fidelity of textual data, allowing the researchers to explore broad and complex contexts, such as national health care systems, with relative computational ease.

To the authors’ knowledge, this is one of the first studies adopting topic modeling in the health sector [56, 57], and the first one focussing on the concept of performance. The interesting results obtained and here discussed suggests that further researches could adopt this methodology to focus on complex issues such as hospitals’ performance.

This study presents some limitations. The first one refers to the methodology itself. One main limitation of topic modeling, and of other so-called bag-of-words approaches to text analysis, is the assumption that the meaning of texts is only conveyed by words’ frequency and co-occurrence within units of analysis. This amounts to ignore the importance of words order, of logical connectors and of other functional words [58].

Another limit of the study is related to context analysed, i.e. the exclusive focus on autonomous public hospitals operating in the Italian health system. Although this last can be considered as a virtuous health system, its governance setting, financing scheme and organizational structure certainly have influenced the results obtained which, due to that, are not entirely generalizable. To overcome such a limitation, future studies may also involve other kinds of healthcare organizations (such as LHA or private hospitals) and provide international benchmarking. Moreover, it could be useful to integrate this study with other sources of information such as scientific articles and grey literature. Further studies may also consider as a level of analysis the regional health systems to check whether hospitals belonging to different systems conceive and address performance differently.

## Conclusions

This article aims at verifying the existence of gaps and differences between literature and practice on the discourse related to hospital performance. In particular, through an analysis of the performance plans of Italian public hospitals, the paper identifies those topics characterising hospital performance as intended by the hospitals’ management. This was then compared, in the discussion section, against public management and healthcare management literature interpretation of public hospital performance.

Answering the first research question of this study – i.e., how is performance defined, conceived, and interpreted in public hospitals practice? – the authors identify and discuss 15 topics. Excluding those related to the regulatory framework, it emerges that today’s performance can be described by a substantial equilibrium of aspects related to structures, outputs, and outcomes and to the ability to take care of the reference population through the integration and coordination with other health system stakeholders. This way to conceive hospital performance is the result of a process that witnessed the growing importance of the topic related to outcomes and population over the last decades.

For what concern the second research question – i.e., to what extent public hospitals embraced or are embracing the theoretical paradigms emerging in the literature with regards to performance – the finding reveals that while hospitals are progressively adopting outcome-based performance management and the population value paradigm, decreasing attention has been devoted to the topic representing collaborative governance and integrated care. In this sense, considering the evolution of epidemiological and governance conditions all over the World, public hospitals’ managers may consider the opportunity to give back priority to such an important aspect also in this kind of documents.

## Supplementary Information

**Additional file 1.** Data source description (pages and documents per hospital per year).

## Data Availability

Data sources are public available in the Italian public hospital webpages and collected in the following repository https://drive.google.com/drive/u/0/folders/1iJaHz3ZkyxDv-UJjIaMdMy3VI98qPT7W. The dataset resulting from the analysis of these documents is available upon request.
