# Supplementary material for: What is the performance in public hospitals? A longitudinal analysis of performance plans through topic modeling
Source: BMC Health Serv Res. 2021 Apr 9;21:326. doi: 10.1186/s12913-021-06332-4 (PMC8033690; doi:10.1186/s12913-021-06332-4)
Supplement: Supplementary file 1 — Additional file 1. Data source description (pages and documents per hospital per year). [file 12913_2021_6332_MOESM1_ESM.docx]

**Additional file 1: Data source description (pages and documents per hospital per year)**

| **Region** | **Hospital** | **2011** | **2012** | **2013** | **2014** | **2015** | **2016** | **2017** | **2018** | **2019** |
| --- | --- | --- | --- | --- | --- | --- | --- | --- | --- | --- |
| I | 1 |  |  |  | 52 | 36 | 32 | 40 | 33 | 42 |
| II | 1 |  |  |  |  |  |  | 105 | 46 | 50 |
|  | 2 |  |  |  | 44 | 46 | 45 | 49 |  | 28 |
|  | 3 |  |  |  |  |  |  |  |  | 81 |
|  | 4 |  |  |  |  | 70 |  |  | 30 | 31 |
| III | 1 |  |  |  |  |  |  | 63 | 36 | 36 |
|  | 2 |  |  |  | 40 |  | 46 | 50 | 52 | 60 |
|  | 3 |  |  |  |  |  |  | 48 |  | 11 |
|  | 4 |  |  |  |  |  |  | 27 |  | 33 |
|  | 5 |  |  |  |  |  |  | 186 | 119 | 44 |
|  | 6 |  |  |  | 39 | 45 |  | 60 | 62 | 73 |
|  | 7 |  |  |  | 70 |  | 47 |  | 41 | 40 |
| IV | 1 |  |  |  |  | 21 | 89 |  | 115 |  |
|  | 2 |  |  | 48 |  | 138 | 78 |  | 107 |  |
|  | 3 |  |  |  |  | 77 | 73 | 72 | 79 |  |
|  | 4 |  |  |  |  | 41 | 67 |  | 95 |  |
| V | 1 |  |  |  |  |  |  |  |  | 47 |
|  | 2 |  |  |  |  |  |  |  | 36 | 36 |
|  | 3 |  |  |  |  |  |  |  |  | 67 |
|  | 4 |  |  |  | 36 |  |  |  |  | 15 |
| VI | 1 |  |  |  |  |  |  |  | 48 | 36 |
|  | 2 |  |  |  |  | 23 |  |  | 25 |  |
| VII | 1 |  | 33 |  | 38 | 48 | 38 | 40 | 40 | 39 |
| VIII | 1 |  |  |  | 45 |  |  | 70 | 59 | 51 |
|  | 2 |  |  |  | 26 | 26 | 41 | 42 | 27 | 25 |
| IX | 1 |  |  |  | 28 | 37 | 24 | 22 |  | 123 |
|  | 2 |  |  |  |  | 28 |  | 33 | 52 | 31 |
|  | 4 |  |  |  |  |  | 126 |  |  | 82 |
| X | 1 |  |  |  |  | 50 |  | 64 |  | 22 |
| XI | 1 |  |  | 32 | 45 | 53 | 50 | 82 | 48 | 44 |
|  | 2 |  |  |  |  |  | 52 | 54 | 59 |  |
|  | 3 |  |  |  |  |  |  | 39 | 50 | 56 |
| XII | 1 |  |  |  |  | 41 | 41 | 39 | 40 | 39 |
|  | 2 |  |  | 18 | 16 | 19 |  | 53 | 44 | 62 |
|  | 3 |  |  |  |  |  |  |  |  | 138 |
|  | 4 | 83 |  | 50 | 50 | 55 | 72 | 76 | 75 | 84 |
|  | 5 |  |  | 32 | 32 | 32 | 40 | 48 | 48 | 46 |
|  | 6 |  |  | 43 |  | 35 | 43 | 44 | 50 | 48 |
|  | 7 |  |  | 25 | 23 |  |  | 57 | 120 | 118 |
|  | 8 | 30 |  |  | 42 |  |  | 41 | 28 | 20 |
| XIII | 1 |  |  |  | 45 | 19 | 44 | 41 | 38 | 30 |
|  | 2 |  |  |  | 16 | 22 | 34 | 43 | 40 | 69 |
|  | 3 |  |  |  | 50 | 51 | 50 | 60 | 55 | 67 |
| XIV | 1 |  |  |  |  |  |  | 61 |  |  |
|  | 2 |  |  |  |  |  |  |  | 67 | 78 |
| XV | 1 |  |  | 36 |  | 64 | 39 | 40 | 38 | 47 |
|  | 2 |  |  |  |  |  |  |  |  | 47 |
| **Average nr of pages (year)** | | **57** | **33** | **36** | **39** | **45** | **53** | **56** | **56** | **52** |
| **Nr of documents (year)** | | **2** | **1** | **8** | **19** | **24** | **22** | **31** | **34** | **40** |
| **Average nr of pages (triennium)** | | **39** | | | **46** | | | **55** | | |
| **Nr of pages (triennium)** | | **430** | | | **2985** | | | **5749** | | |
| **Nr of words (triennium)** | | **130,290** | | | **892,515** | | | **1,782,190** | | |
